# Improving Yield and Thermostability of PETase as a Maltose Binding Protein Fusion in the Periplasm of *Escherichia coli*

**DOI:** 10.3390/ijms27072962

**Published:** 2026-03-25

**Authors:** Jiin Kwon, Seri Koh, Soyeon Jang, Huiwon Cho, Minjeong Shin, Heehyeon Jeon, Suah Cho, Sooyeon Jung, Rang Choi, Eunsoo Lee, Yeeun Kim, Wonchull Kang

**Affiliations:** 1 Department of Chemistry, College of Natural Sciences, Soongsil University, Seoul 06978, Republic of Koreaseri2323@daum.net (S.K.); chlfkd0504@naver.com (R.C.);; 2 Department of Green Chemistry and Materials Engineering, Soongsil University, Seoul 06978, Republic of Korea

**Keywords:** poly(ethylene terephthalate) hydrolase, PETase, *Ideonella sakaiensis*, protein engineering, maltose-binding protein, periplasmic translocation

## Abstract

Polyethylene terephthalate (PET) waste accumulation requires sustainable recycling alternatives. While *Ideonella sakaiensis* PETase offers a green solution, its industrial application is hindered by low solubility and poor thermostability. In this study, we systematically evaluated the synergistic effects of maltose-binding protein (MBP) fusion and periplasmic translocation strategies to optimize PETase production in *Escherichia coli*. Our results demonstrate that MBP acts as a potent solubilizing partner for PETase, with the cytosolic MBP–PETase variant achieving a high purification yield of 8.4 mg per gram of wet cell weight–a significant improvement over the PelB–PETase control (1.1 mg per gram of wet cell weight). Furthermore, the periplasmic MalE–MBP–PETase construct provided an optimal intermediate compromise between the yield, thermal stability, and catalytic activity by leveraging the oxidative environment of the periplasm for critical disulfide bond formation. Although PelB–PETase exhibited higher specific activity, its low yield limits industrial scalability. This study establishes a robust plug-and-play platform for high-throughput PET depolymerization, providing a foundational step toward a circular plastic economy.

## 1. Introduction

Polyethylene terephthalate (PET) has become an indispensable material in modern industry due to its excellent mechanical properties, chemical resistance, and versatility. Consequently, it dominates global plastic production, primarily distributed across beverage bottles, food packaging, and synthetic textiles [1,2]. Despite its utility, the massive accumulation of PET waste poses a severe environmental threat, as it persists in landfills and marine ecosystems for centuries [2,3]. Currently, mechanical recycling is the most widely adopted method; however, it suffers from downcycling, where the structural integrity of the polymer degrades with each cycle, eventually rendering it unrecyclable [2]. Furthermore, contamination with other plastic types or organic matter often complicates the process. Thermal incineration, another common disposal route, offers energy recovery but generates significant greenhouse gases and toxic byproducts [4]. Therefore, there is an urgent need for a sustainable green alternative that can support a truly circular plastic economy.

The discovery of *Ideonella sakaiensis 201-F6* and its specialized enzyme, PETase, marked a landmark shift in biocatalysis [5,6]. Unlike traditional chemical hydrolysis, which requires high temperatures and harsh pH conditions, PETase operates under ambient conditions to depolymerize PET into its constituent monomers, i.e., terephthalic acid (TPA) and ethylene glycol (EG) [5]. These monomers can be purified and re-polymerized into virgin-grade PET, facilitating a closed-loop recycling system. However, despite its potential, wild-type PETase faces critical limitations that hinder its industrial application. It exhibits low expression yields in common microbial hosts and possesses suboptimal thermostability, often denaturing at temperatures close to the glass transition temperature of PET, where the polymer chains become more accessible to enzymatic attack [7]. Furthermore, its catalytic activity against highly crystalline PET is insufficient for rapid industrial processing [8]. To overcome these hurdles, prior protein engineering efforts have yielded variants with enhanced thermostability and kinetic parameters [9]. Notable examples include Mipa-P and Kubu-P, as identified through extensive homolog searching and directed evolution, which outperform wild-type activity under industrially relevant conditions [10].

A fundamental challenge that remains under-addressed is the efficient production of these enzymes in microbial factories such as *Escherichia coli* (*E. coli*). PETase adopts a classic α/β-hydrolase fold but uniquely relies on two essential disulfide bonds (Cys203–Cys239 and Cys237–Cys289) [11,12,13,14,15]. These bonds are crucial for maintaining the integrity of the catalytic pocket and the dynamics of the lid domain, which is essential for substrate binding and access [11,12,13,14,15]. In the reducing environment of the *E. coli* cytoplasm, these disulfide bonds form inefficiently, leading to protein misfolding, the formation of insoluble inclusion bodies, and low functional yields.

To address these folding and solubility issues, various molecular biology strategies have been employed [9]. Periplasmic translocation via Sec-dependent signal peptides, such as PelB and MalE, exploits the oxidative environment of the *E. coli* periplasm, which facilitates the proper formation of native disulfide bonds [16,17,18,19]. Additionally, the use of maltose-binding protein (MBP) as a fusion tag has proven effective. MBP, a 42 kDa chaperone-like protein, enhances the solubility of its fusion partner by shielding hydrophobic regions and accelerating the folding kinetics [20].

While both periplasmic export and MBP solubilization are known techniques, their synergistic effect on PETase production remains largely unexplored. Different signal peptides exhibit varying degrees of efficiency; for instance, the MalE signal sequence is known for high-fidelity export. Furthermore, the trade-off between periplasmic recovery (via osmotic lysis) and total cell lysis (via sonication) requires careful optimization to preserve protein integrity and prevent protease degradation.

In this study, we hypothesized that a MalE–MBP–PETase tripartite system would maximize the synergy between MBP-driven folding and oxidative disulfide maturation in the periplasm. By comparing this system against cytosolic MBP fusions and unfused periplasmic PelB benchmarks, we aimed to establish a robust, plug-and-play platform for PETase biocatalysis. Our approach focuses on balancing the yield, stability, and kinetics without the immediate need for extensive mutagenesis. The results demonstrate that periplasmic MBP–PETase is an industrially viable candidate, capable of producing gram-scale enzyme from simple shake-flask cultures for pilot-scale PET depolymerization. This work provides a foundation for high-throughput recycling processes, moving toward a sustainable and circular plastic economy.

## 2. Results

### 2.1. Design of PETase Variants

**Figure 1:**
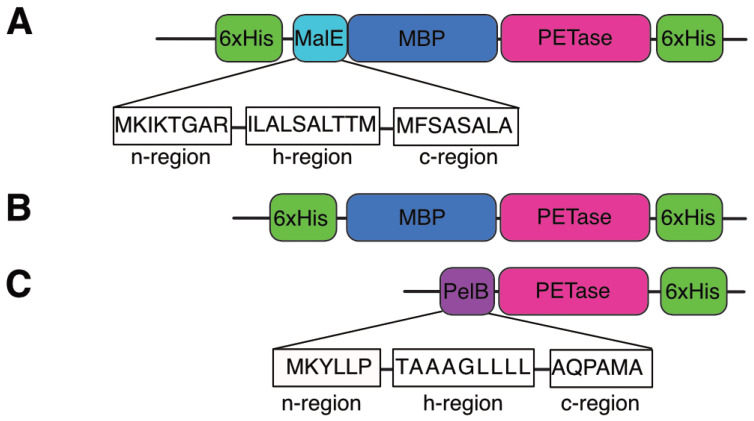
Schematic representation of the PETase variants. (**A**) PETase fused to maltose-binding protein (MBP) containing the N-terminal MalE signal peptide for periplasmic expression. (**B**) PETase fused to MBP without a signal peptide for cytosolic expression. (**C**) PETase fused to the PelB signal peptide.

Schematic diagrams of the engineered PETase variants are shown in Figure 1. To investigate the impact of the fusion partners on the protein expression, solubility, and stability, maltose-binding protein (MBP) was N-terminally fused to PETase from *Ideonella sakaiensis 201-F6*. PETase naturally contains two structurally critical disulfide bonds (Cys203–Cys239 and Cys237–Cys289), which form inefficiently in the reducing environment of the *E. coli* cytoplasm. Therefore, strategies to translocate the enzyme to the oxidative periplasm were employed to promote proper folding and to enhance the thermostability. Two MBP fusion constructs were designed: (A) MalE signal peptide–MBP–PETase, leveraging the MalE signal peptide sequence for Sec-dependent periplasmic export followed by osmotic lysis recovery, and (B) MBP–PETase without a signal peptide, remaining cytosolic with sonication lysis (Figure 1A,B). As a control for periplasmic translocation via the Sec pathway, PelB signal peptide–PETase was included (Figure 1C). All constructs incorporated an N-terminal His-tag (or dual His-tags at both termini where feasible) to facilitate Ni-NTA affinity purification and ensure high purity for downstream functional assays, consistent with the established protocols for PETase engineering. These designs systematically test the combined effects of fusion solubilization tags and the compartmental localization of PETase performance.

### 2.2. Expression of PETase Variants

**Figure 2:**
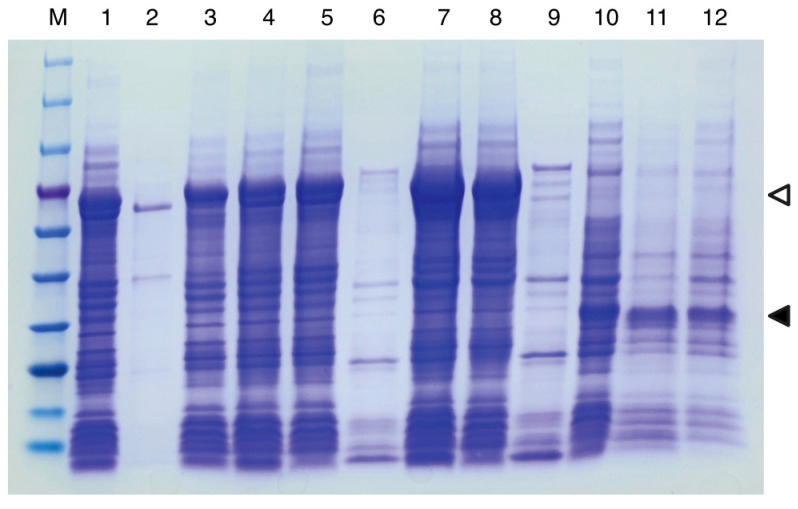
SDS-PAGE analysis of PETase variant expression and solubility. The gel shows the total cell lysate, soluble supernatant, and insoluble precipitate fractions for each extraction; lane 1–3, osmotic shock extraction of MalE–MBP–PETase; lane 4–6, sonication extraction of MalE–MBP–PETase; lane 7–9, sonication extraction of MBP–PETase; lane 10–12 sonication extraction of PelB–PETase. M indicates the protein molecular weight marker (180, 140, 100, 75, 60, 45, 35, 25, 15 and 10 kDa; LPS Solution, Korea). Empty triangles represent the target band for MBP–PETase (and MalE–MBP–PETase), and filled triangles represent the target band for PelB–PETase.

All PETase variants were expressed under the T7 promoter system with 0.5 mM isopropyl β-D-1-thiogalactopyranoside (IPTG) induction. The expression and solubility levels were assessed via Coomassie-stained SDS-PAGE analysis of total cell lysates, soluble supernatants, and insoluble precipitates (Figure 2). MBP-fused PETase showed robust expression after 3 h and overnight induction at 289 K compared with the uninduced controls. The MalE–MBP–PETase construct demonstrated superior periplasmic localization via osmotic lysis, yielding predominantly soluble proteins in the supernatant fraction with high purity. In contrast, sonication lysis of the same construct, MBP–PETase, and PelB signal peptide–PETase revealed varying solubility profiles, with MBP fusions generally improving the soluble fractions relative to the PelB control. Target bands appeared at the expected molecular weights, confirming the successful expression of full-length fusions without major truncation. Due to the negligible yield of PelB–PETase obtained via osmotic lysis, this method was excluded from further comparative analysis. The precise quantitative efficiency of soluble protein partitioning for each construct was subsequently determined by measuring the final purified protein yield (mg per gram of wet cell weight), which is detailed in Section 2.3 and Table 1. These results indicate that MBP fusion enhances cytosolic solubility, while signal peptide-directed periplasmic export further improves the recovery for the MalE–MBP–PETase variant.

### 2.3. Purification of PETase Variants

**Figure 3:**
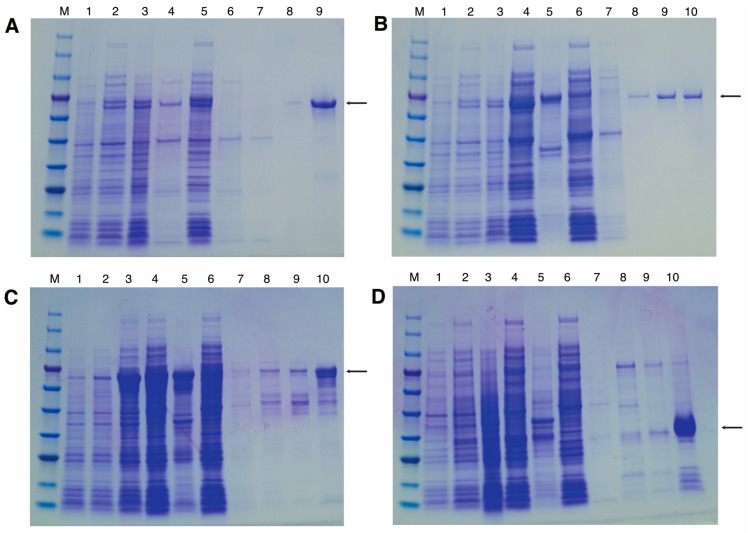
Ni-NTA affinity purification of PETase variants. Coomassie brilliant blue-stained SDS-PAGE gels showing the expression, solubility, and purification steps from *E. coli.* Each panel corresponds to a specific construct from Figure 1: (**A**) MalE–MBP–PETase (osmotic lysis); (**B**) MalE–MBP–PETase (sonication lysis); (**C**) MBP–PETase (sonication lysis); (**D**) PelB–PETase (sonication lysis). Lanes: M, protein molecular weight marker (see Figure 2 for details); 1, uninduced; 2–3, induced for 3 h and overnight; 4–5, soluble supernatant and insoluble precipitate fraction; 6–7, flow-through and wash fractions; 8–10, elution fractions. Arrowheads indicate the purified target PETase bands.

**Figure 4:**
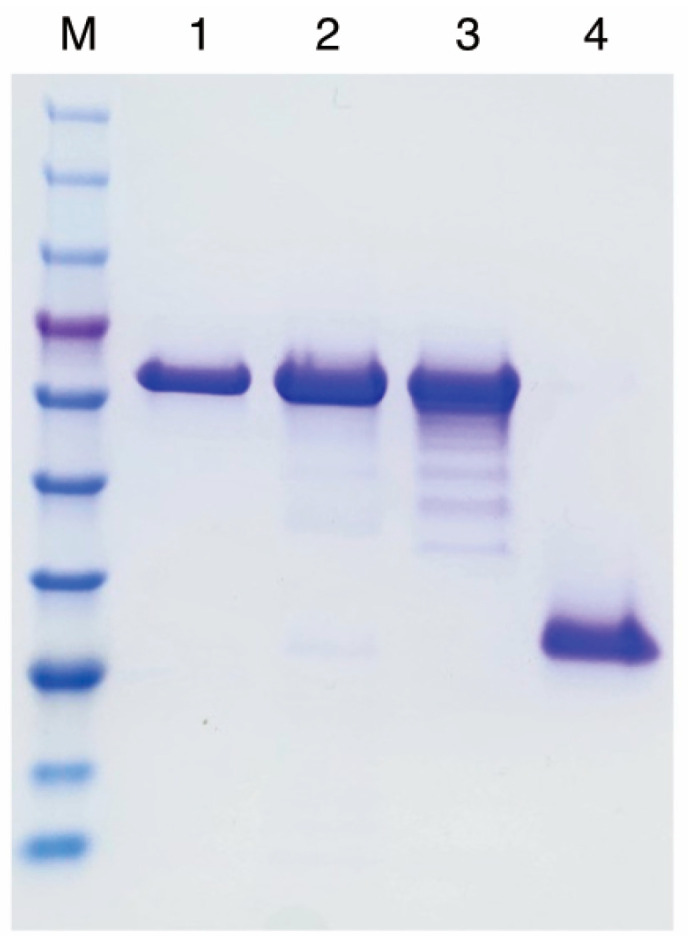
Comparison of final purified PETase variants. Coomassie brilliant blue-stained SDS-PAGE of purified proteins collected from Ni-NTA elution fractions. Lanes: M, protein molecular weight marker (see Figure 2 for details); 1, MalE–MBP–PETase (osmotic lysis); 2, MalE–MBP–PETase (sonication lysis); 3, MBP–PETase (sonication lysis); 4, PelB–PETase (sonication lysis). All variants were purified to near homogeneity for the subsequent experiments.

Purified PETase variants were obtained via Ni-NTA affinity chromatography from clarified lysates, with the expression, solubility, and purification progress monitored using Coomassie-stained SDS-PAGE (Figure 3 and Figure 4). For the MalE–MBP–PETase construct extracted by osmotic lysis (Figure 3A), strong expression was evident after overnight induction, with most protein partitioning to the soluble supernatant. However, the duration of osmotic lysis compromised the final yield, yielding 3 mg per gram of wet cell weight. Ni-NTA purification produced clean eluate fractions, confirming effective His-tag capture. Sonication lysis of MalE–MBP–PETse (Figure 3B) and MBP–PETase (Figure 3C) showed comparable expression level but substantially higher yields, particularly for MBP–PETase without the MalE signal peptide. The yields were 6.2 mg per gram of wet cell weight for MalE–MBP–PETase and 8.4 mg per gram of wet cell weight for MBP–PETase. In contrast, the PelB–PETase constructs exhibited a low expression level (Figure 3D) and yielded only 1.1 mg per gram of wet cell weight. Osmotic lysis of PelB signal peptide–PETase was attempted several times, but it showed insufficient yield. The final purified proteins from Ni-NTA eluates demonstrated high purity across all variants (Figure 4). Only MBP–PETase showed minor smearing/laddering, potentially indicating limited protease degradation (Figure 3C). Overall, MBP fusion substantially enhanced the yield compared to the PelB signal peptide control, with cytosolic MBP–PETase providing optimal recovery.

### 2.4. Thermostability Comparison of PETase Variants

**Figure 5:**
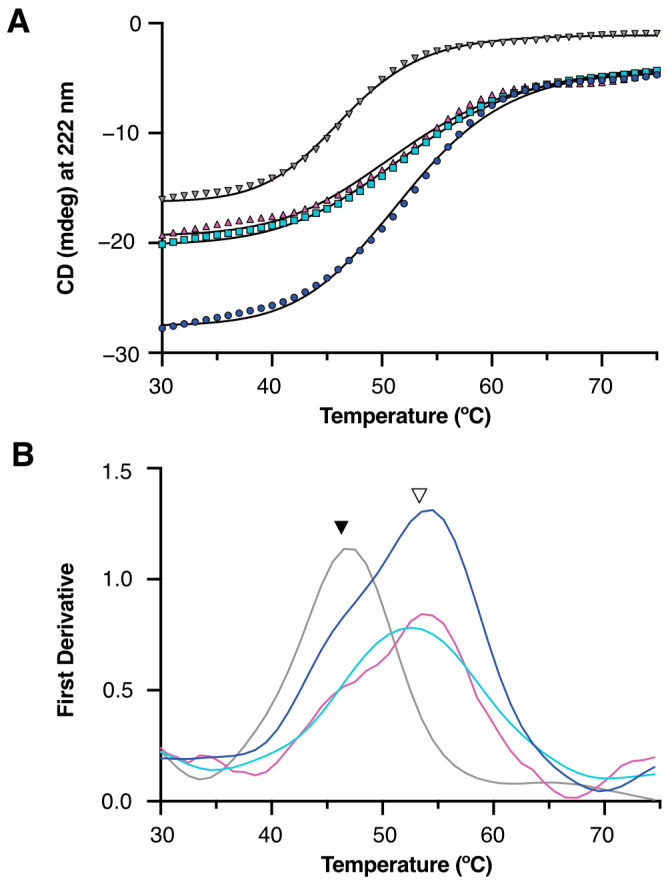
Thermal stability of the purified PETase variants assessed via circular dichroism (CD) spectroscopy. (**A**) Far-UV CD thermal denaturation curves monitored at 222 nm. Protein unfolding is represented as the mean residue ellipticity versus temperature. Blue, MalE–MBP–PETase (osmotic lysis); cyan, MalE–MBP–PETase (sonication lysis); magenta, MBP–PETase (sonication lysis); grey, PelB–ETase (sonication lysis). Solid lines represent nonlinear sigmoidal fits used to determine the melting temperatures (T_m_). (**B**) First derivative plots of the thermal denaturation curves in (**A**), with peaks indicating the T_m_ values for each variant. Color coding matches panel (**A**). Filled inverted triangles represent the T_m_ of PETase; empty inverted triangles represent the T_m_ of MBP–PETase.

The thermal stability of the purified PETase variants was assessed using far-UV circular dichroism (CD) spectroscopy, monitoring the unfolding at 222 nm (Figure 5). Non-linear sigmoidal fits of the thermal denaturation curves yielded melting temperatures (T_m_) as follows: periplasmic MalE–MBP–PETase (osmotic lysis), 52.0 °C; cytosolic MalE–MBP–PETase (sonication lysis), 52.4 °C; cytosolic MBP–PETase, 51.5 °C; and PelB–PETase (sonication lysis), 46.7 °C. First derivative plots confirmed these inflection points, with the peaks clearly resolving T_m_ values for each construct. MBP fusion substantially improved the thermostability across all variants relative to the PelB–PETase control, likely due to enhanced folding and reduced aggregation in the cytosolic environments. Periplasmic translocation via MalE signal peptide provided no additional thermal benefit over cytosolic MBP fusions, as both MalE–MBP–PETase preparations exhibited identical T_m_ values. These results demonstrate that MBP solubilization tagging confers robust thermal stability to PETase, independent of the cellular compartment. However, because MBP is a large (~42 kDa) and inherently stable protein, it is important to consider that the observed CD signal and resulting thermal denaturation curve (Tm ~53.5 °C) may be heavily dominated by the global unfolding of the MBP tag itself. This structural robustness of the fusion partner could potentially mask the independent unfolding events of the smaller PETase domain or artificially stabilize the fusion protein.

### 2.5. Catalytic Activity Comparison of PETase Variants

**Figure 6:**
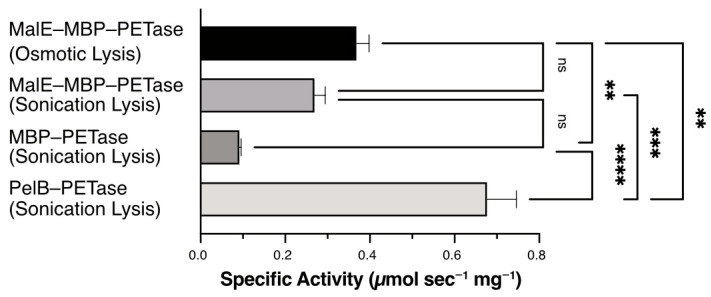
Specific enzymatic activities of purified PETase variants. Bar graphs showing PETase activity on the substrate *p*-nitrophenyl acetate (*p*-NPA). Data represent the means ± standard error of the means (SEM) from three independent replicates (*n* = 3). Statistical significance was determined by one-way ANOVA followed by Tukey’s multiple comparisons test (** *p* < 0.01, *** *p* < 0.001, **** *p* < 0.0001, ns: not significant).

Specific enzymatic activities of the purified PETase variants were measured using *p*-nitrophenyl acetate (*p*-NPA) as the substrate (Figure 6). PelB–PETase exhibited the highest activity at 0.68 μmol s^−1^ mg^−1^, substantially outperforming all MBP fusion constructs. Although sonication is a whole-cell lysis technique, it effectively releases the periplasmic fraction where PelB-PETase has successfully translocated and formed proper disulfide bonds, which explains its high catalytic activity despite poor recovery via osmotic shock. The periplasmic MalE–MBP–PETase (osmotic lysis) showed 0.37 μmol s^−1^ mg^−1^. Interestingly, the MalE–MBP–PETase recovered via sonication displayed a statistically comparable specific activity of 0.27 μmol s^−1^ mg^−1^, while the cytosolic MBP–PETase displayed a significantly lower activity at 0.09 μmol s^−1^ mg^−1^. From an industrial perspective, this lack of significant difference in activity between the extraction methods is highly advantageous. It indicates that the sonication lysis of MalE–MBP–PETase can achieve a twofold higher yield (6.2 mg g^−1^) without a statistically significant compromise in catalytic performance compared to osmotic lysis. This demonstrates that the construct can withstand harsh and highly scalable mechanical disruption, which is strongly preferred over time-consuming and low-yielding osmotic shock protocols for large-scale enzyme production. Although PelB–PETase demonstrated approximately twofold higher specific activity than periplasmic MalE–MBP–PETase, the latter provided a threefold higher yield (3 mg g^−1^ wet cell weight vs. 1.1 mg g^−1^ wet cell weight). Periplasmic translocation via MalE signal peptide showed improved activity over cytosolic MBP fusions, likely due to proper disulfide bond formation in the oxidative periplasm, synergizing with MBP solubilization. However, the unfused PelB–PETase control achieved maximal catalysis despite a low yield, indicating that fusion tags may impose steric constraints on the active site despite enhancing expression. A comprehensive quantitative comparison of the purification yields, specific activities, and melting temperatures for all evaluated constructs is summarized in Table 1.

### 2.6. Kinetic Parameters of Periplasmic MBP-Fused PETase

**Figure 7:**
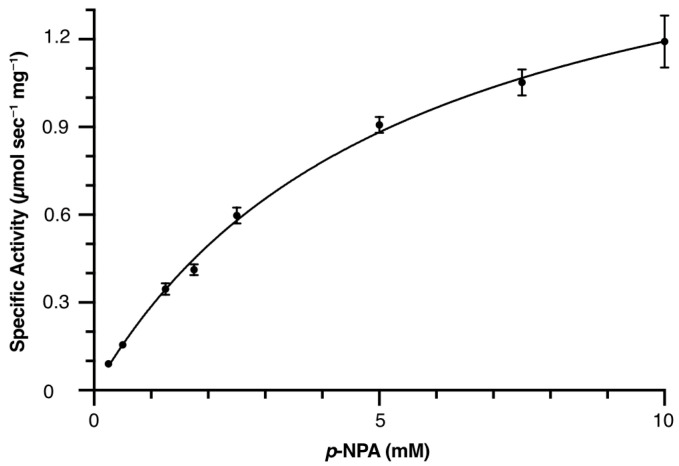
Michaelis–Menten kinetics of purified MalE–MBP–PETase (osmotic lysis) using *p*-nitrophenyl acetate (*p*-NPA) as a substrate. The specific activity is plotted against *p*-NPA concentration. Data points represent the means ± standard error of the mean (SEM, *n* = 3 replicates). The curve represents a nonlinear regression fit to the Michaelis–Menten equation used to derive kinetic parameters.

Michaelis–Menten kinetics parameters for the periplasmic MalE–MBP–PETase (osmotic lysis) were determined using *p*-NPA as substrate (Figure 7). The non-linear regression fits of the initial velocity data yielded 1.84 μmol s^−1^ mg^−1^ of V_max_, 5.4 mM of K_m_, 136.4 s^−1^ of k_cat_, and 2.5 × 10^4^ M^−1^ s^−1^ of catalytic efficiency. Compared to values for native PETase [21], the periplasmic MBP fusion maintained comparable catalytic performance despite the large N-terminal tag. The modest increase in K_m_ suggests minor substrate access hindrance, while high k_cat_ indicates preserved active-site dynamics. The balanced kinetics of this construct, combined with its superior yield and thermostability, position periplasmic MalE–MBP–PETase as optimal for biocatalytic applications.

## 3. Discussion

In this study, we systematically evaluated the synergistic effects of maltose-binding protein (MBP) fusion and periplasmic translocation strategies to optimize the expression, solubility, and thermal stability of *Ideonella sakaiensis* PETase. Our data highlight that while PETase is inherently difficult to produce in a soluble form within the *E. coli* cytoplasm, the choice of fusion partner and signal peptide significantly dictates the functional yield and structural integrity of the enzyme.

Our results demonstrate that MBP acts as a potent solubilizing partner for PETase. The cytosolic MBP–PETase variant achieved the highest purification yield of 8.4 mg per gram of wet cell weight, representing a significant improvement over the PelB–PETase control, which yielded only 1.1 mg per gram of wet cell weight. This enhancement is likely due to the chaperone-like activity of MBP, which facilitates the proper folding of the PETase domain and prevents the formation of inclusion bodies that typically occur in the reducing environment of the cytoplasm. The technological advantage of using the MalE signal peptide lies in its synergistic compatibility with the MBP tag. Because both elements are derived from the same *malE* gene system, they facilitate a highly efficient translocation process. The periplasmic MalE–MBP–PETase construct not only achieved a high purification yield but also maintained optimal catalytic kinetics. This suggests that the MalE-mediated export process provides a superior oxidative environment necessary for the formation of critical disulfide bonds, which are essential for maintaining the structural integrity and the activity of PETase. Although we did not perform direct analytical confirmation of disulfide bond maturation (e.g., via mass spectrometry), the significantly improved specific activity of our periplasmic MalE–MBP–PETase construct compared to the cytosolic MBP fusions serves as a strong functional indicator of proper structural folding. The enzymatic activity in this study was evaluated using *p*-NPA, which is a widely utilized and robust screening method for determining basic esterase kinetic parameters and serves as an effective comparative benchmark; however, it does not represent the heterogeneous crystalline surface of natural PET plastic. Future studies utilizing PET film degradation or PET nanoparticle assays will be necessary to confirm the actual PET degradation efficiency.

Interestingly, while the fusion tags improved the yield, they also introduced considerations regarding catalytic access. PelB–PETase recovered via whole-cell sonication exhibited the highest specific activity at 0.68 μmol s^−1^ mg^−1^, substantially outperforming all MBP fusion constructs regarding raw kinetics. This indicates that the presence of a large fusion protein such as MBP may cause slight steric hindrance at the active site. However, the low overall yield of the unfused PelB–PETase makes it less viable for large-scale applications compared with the fusion variants. Ultimately, the periplasmic MalE–MBP–PETase construct emerged as the “optimal” platform. This designation is strictly defined as an intermediate compromise: while it does not possess the absolute highest activity (PelB–PETase), it provides the best functional balance. It delivers an industrially acceptable recovery yield (up to 6.2 mg g^−1^ via sonication) while retaining a functionally viable specific activity. Furthermore, the statistical equivalence in specific activity between its osmotic and sonicated preparations demonstrates structural robustness. The ability to utilize rapid, scalable sonication without sacrificing enzymatic integrity is a critical advantage for the cost-effective pilot-scale production of PETase. By combining solubilization tagging with compartmentalized export, this construct bridges the gap between high-yield protein production and functional enzymatic integrity. These findings provide a robust and plug-and-play platform for future industrial biocatalyst engineering toward circular plastic economies.

## 4. Materials and Methods

### 4.1. Cloning

The coding sequences of *Ideonella sakaiensis 201-F6* PETase (*Is*PETase) were derived from pCJ190, a gift from Dr. Gregg Beckham (Addgene plasmid # 162667; http://n2t.net/addgene:162667; RRID: addgene_162667; accessed on 16 August 2022) [22]. The PETase gene (residues 28–290) was cloned into the pET-22b(+) (Novagen, St. Louis, MO, USA) vector, which encodes an N-terminal pelB signal sequence and a C-terminal 6xHis-tag. For maltose-binding protein (MBP) fusion, *Is*PETase was cloned into pVFT4S (a gift from Dr. Kyeong Kyu Kim at Sungkyunkwan University), featuring an N-terminal 6xHis-tag and MBP. *Is*PETase was also inserted into pVFT5S (a gift from Dr. Kyeong Kyu Kim at Sungkyunkwan University), which includes an N-terminal 6xHis-tag, MBP, malE signal sequences, and a C-terminal 6xHis-tag. Nucleotide sequences of the recombinant plasmids were verified via sequencing (Bionics, Seoul, Republic of Korea). All cloning enzymes were obtained from New England Biolabs (Ipswich, MA, USA).

### 4.2. Overexpression and Purification of IsPETase

Recombinant plasmids were transformed into *Escherichia coli* Rosetta 2 (DE3) (Novagen, USA). MBP fusion *Is*PETase was overexpressed at 289 K for 18 h in 2× YT medium containing 30 μg mL^−1^ of kanamycin. pelB signal/6xHis-IsPETase was overexpressed at 298 K for 18 h in LB medium containing 100 μg mL^−1^ of ampicillin. Overexpression was induced with 0.5 mM isopropyl β-D-1-thiogalactopyranoside (IPTG) at 0.6 of OD_600_. Cells were harvested via centrifugation at 5000× *g* for 20 min and stored at 203 K. For periplasmic extraction via osmotic shock, the method from the previous research was employed [23]. Briefly, the cell paste was resuspended in the working buffer (20 mM Tris-HCl (pH 8.0), 100 mM sodium chloride, 10 mM imidazole, and 5% (*v/v*) glycerol) supplemented with 25% (*w/v*) sucrose, followed by stirring on ice for 30 min. The cells were then collected by centrifugation at 277 K and suspended in the working buffer, followed by stirring on ice for 30 min. The cells were pelleted again by centrifugation. The supernatant, which is the osmotic shock fluid, was filtered through a 0.45 μm polyethersulfone membrane (Sartorius, Göttingen, Germany) and applied to Ni Sepharose 6 Fast Flow (Cytiva, Marlborough, MA, USA) equilibrated with the working buffer. The column was washed with a 10-column volume of the working buffer supplemented with 50 mM imidazole and eluted with 250 mM imidazole. The fractions containing PETase were concentrated using Vivaspin (Sartorius, Germany) to a final concentration of 2.2 mg mL^−1^. For cell disruption by ultrasonication, the resuspended cells in the working buffer were lysed by sonication with a VCX500 ultrasonic processor (Sonics & Materials, Newtown, CT, USA) and pelleted by centrifugation. The supernatant was filtered through a 0.45 μm polyethersulfone membrane (Sartorius, Germany), and the above procedures were applied for purification.

### 4.3. Enzyme Assay for p-Nitrophenyl Acetate (p-NPA) Hydrolysis

The assay method was employed, according to the previous research [5,21]. Briefly, all measurements were performed in 50 mM sodium phosphate buffer (pH 7.5) and 100 mM sodium chloride at 30 °C using an Ultrospec 8000 UV-VIS Spectrophotometer (Cytica, USA). *p*-NPA solution was prepared in DMSO at a concentration of 250 mM, before measurement. The production of *p*-NP by the hydrolysis of *p*-NPA (MilliporeSigma, St. Louis, MO, USA) was monitored at 405 nm for 30 s. The reaction in the total volume of 500 μL in the quartz cuvette was initiated by adding enzyme stock solution to a final concentration of 10 nM. For background data, the volume of the enzyme was replaced by the buffer. Three independent measurements were performed. After the concentration of pNP was calculated using the extinction coefficient (ε_405_ = 18,400 M^−1^ cm^−1^), the enzyme kinetics were analyzed using GraphPad Prism version 11.0.0 (GraphPad Software, Boston, MA, USA). Differences between the experimental groups were analyzed using one-way analysis of variance (ANOVA). To identify specific differences between pairs of variants, Tukey’s post hoc test was applied. A *p*-value of less than 0.05 was considered statistically significant.

### 4.4. Circular Dichroism Spectroscopy

Protein was dialyzed into the buffer containing 50 mM sodium phosphate (pH 8.0) and 5% (*v/v*) glycerol and concentrated to achieve a final concentration of 0.8 mg mL^−1^. The CD spectra of thermal denaturation were recorded at 222 nm on a J-1500 Circular Dichroism Spectrometer (JASCO, Tokyo, Japan). The initial temperature was 25 °C and increased to 95 °C with an interval of 1.0 °C for 1 min. The data were plotted and analyzed using GraphPad Prism version 11.0.0 (GraphPad Software, USA). Due to instrument accessibility constraints, the thermal denaturation curve for each variant was obtained from a single representative measurement (*n* = 1) comprising 51 temperature points. The melting temperature (T_m_) was determined from the inflection point of a 4-parameter logistic (4PL) nonlinear regression model. The statistical reliability of the data was confirmed by the high goodness-of-fit (R^2^ > 0.99) and is reported as the T_m_ alongside the 95% profile likelihood confidence intervals (95% CI).

## Figures and Tables

**Table 1:** Quantitative comparison of the efficiency and properties of purified PETase variants.

Construct	Lysis Method	Yield (mg/g Wet Cell Weight)	Specific Activity (μmol s^−1^ mg^−1^)	Melting Temperature (T_m_, °C) [95% CI]
MalE–MBP–PETase	Osmotic	3.0	0.37	52.0 [51.7–52.4]
MalE–MBP–PETase	Sonication	6.2	0.27	52.4 [52.0–52.9]
MBP–PETase	Sonication	8.4	0.09	51.5 [50.9–52.2]
PelB–PETase	Sonication	1.1	0.68	46.7 [46.2–46.7]

## Data Availability

The original contributions presented in this study are included in the article. Further inquiries can be directed to the corresponding author.
